# Diagnostic and treatment dilemma during the coronavirus disease 2019 pandemic: a primary pulmonary lymphoma presenting as a cavitary mass in a patient with coronavirus disease 2019: a case report

**DOI:** 10.1186/s13256-022-03745-5

**Published:** 2023-01-13

**Authors:** Fatima Wong, Megan Doyle-McClam, Spencer Pugh, Tina Dudney, Michael McCormack, Jared Kravitz

**Affiliations:** Division of Pulmonary and Critical Care Medicine, Department of Medicine, Graduate School of Medicine, University of Tennessee Health Sciences Center Knoxville, Knoxville, TN USA

**Keywords:** COVID-19, Hodgkin lymphoma, Cavitary pulmonary lesion, Bronchoscopy, Percutaneous needle biopsy, Case report

## Abstract

**Background:**

A radiological finding of a cavitary pulmonary lesion in a patient acutely infected with severe acute respiratory syndrome coronavirus-2 early during the coronavirus disease 2019 pandemic created a diagnostic and treatment dilemma, as invasive procedures with bronchoscopy and percutaneous needle lung biopsy posed an infection hazard to healthcare workers due to the associated risk of viral aerosolization. Available guidelines recommended delay of non-emergent procedures, but timely proceeding with those deemed urgent provided appropriate personal protective equipment and negative pressure isolation were available and exposure risk was not excessive. Thoughtful consideration by clinicians was required to avoid delay in diagnosis of a potential new malignancy and prevent unnecessary healthcare worker exposure to the virus. Additionally, acute severe acute respiratory syndrome coronavirus-2 infection in patients with malignancy complicated timing of oncologic treatment.

**Case presentation:**

A 26-year-old otherwise healthy Caucasian male initially presented with an enlarging right upper lobe cavitary pulmonary lesion despite antimicrobial therapy. During his hospitalization and evaluation, the patient was found to be acutely infected with severe acute respiratory syndrome coronavirus-2 without hypoxia or viral pneumonia. Bronchoscopy was deemed too high risk for viral aerosolization and healthcare worker infection. He underwent computed-tomography-guided percutaneous needle biopsy of the lesion by interventional radiology while on mechanical ventilation after elective intubation by anesthesiology. Biopsy revealed classic Hodgkin lymphoma consistent with primary pulmonary Hodgkin lymphoma. After collaboration with oncology, his treatment with combined chemotherapy and immunotherapy was delayed for 3 weeks following diagnosis to allow for viral clearance.

**Conclusion:**

A careful multidisciplinary strategy is required to expeditiously diagnose and treat aggressive cancers of the respiratory tract in patients acutely infected with severe acute respiratory syndrome coronavirus-2 while observing practices to prevent healthcare worker infection during the ongoing coronavirus disease 2019 pandemic.

## Introduction

Cases of primary pulmonary Hodgkin lymphoma (PPHL), in which the predominant involvement of the Hodgkin lymphoma (HL) occurs in the lung with no or minimal nodal involvement, are rare with most described as case reports [1]. PPHL presenting as a cavitary lesion is even more unusual, representing less than 1% of cases [2]. A cavitary pulmonary lesion has various possible infectious, inflammatory, and malignant etiologies; and diagnosis requires an invasive tissue biopsy. During the coronavirus disease 2019 (COVID-19) pandemic, non-emergent procedures posing a high risk for viral aerosolization and infection of hospital personnel, including bronchoscopy and percutaneous needle lung biopsy, were not advised in patients infected with severe acute respiratory syndrome coronavirus-2 (SARS-CoV-2). We present a case of a young man with a right upper lobe cavitary pulmonary lesion ultimately diagnosed as PPHL. During his evaluation he became acutely infected with SARS-CoV-2, which complicated decisions regarding timely tissue biopsy for diagnosis and safely initiating treatment.

## Case

Early in the COVID-19 pandemic in the USA, a 26-year-old previously healthy Caucasian male initially presented to our pulmonary clinic with three months of symptoms including a productive cough, fever, night sweats, myalgias, anorexia, and weight loss. He was earlier treated with antibiotics prescribed by an urgent care clinic (facilities with providers who treat acute, non-life-threatening medical problems). Despite mild initial improvement, his symptoms persisted. He denied chest pain, pleurisy, hemoptysis, joint swelling or stiffness, Raynaud’s phenomena, or rashes. He denied recent travel or sick contacts, prior exposures to persons infected with active tuberculosis, or personal use of tobacco, alcohol, or illicit drugs. He had no chronic medical conditions. He recently enlisted in the US Navy.

**Fig. 1:**
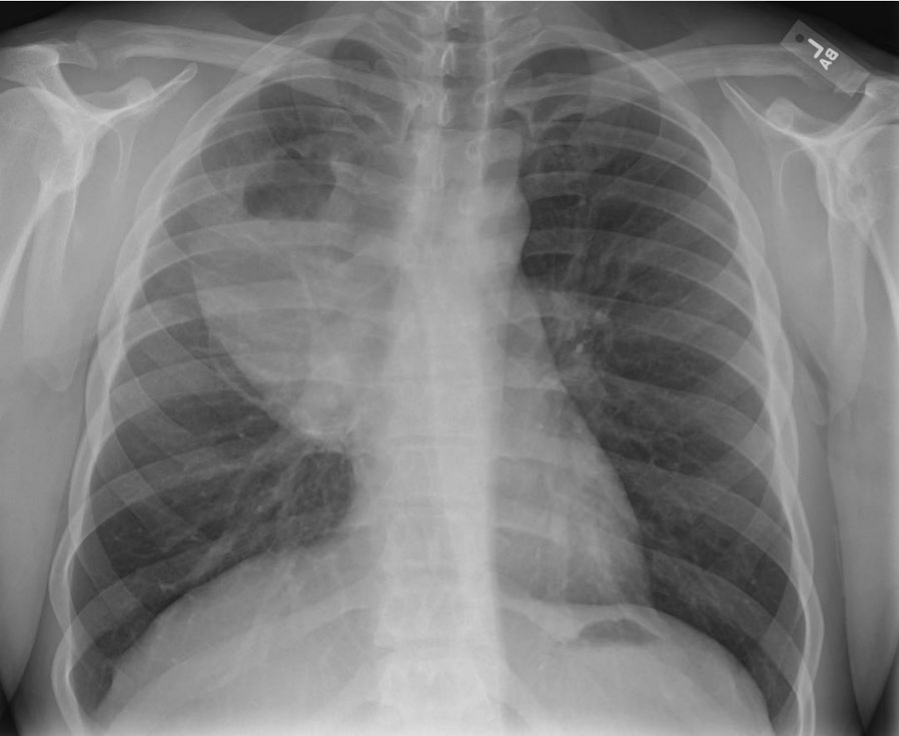
Initial chest X-ray (PA view). Right upper lobe cavitary lesion

**Fig. 2:**
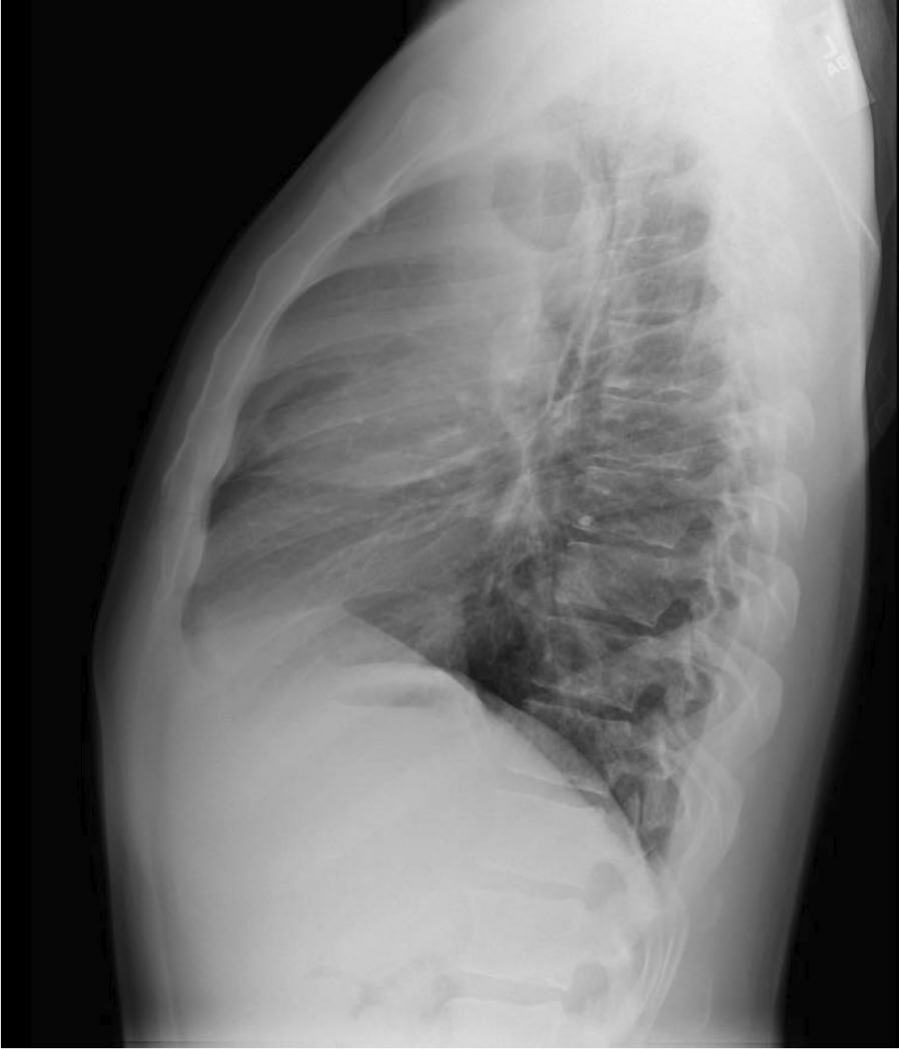
Initial chest X-ray (lateral view). Right upper lobe cavitary lesion

**Fig. 3:**
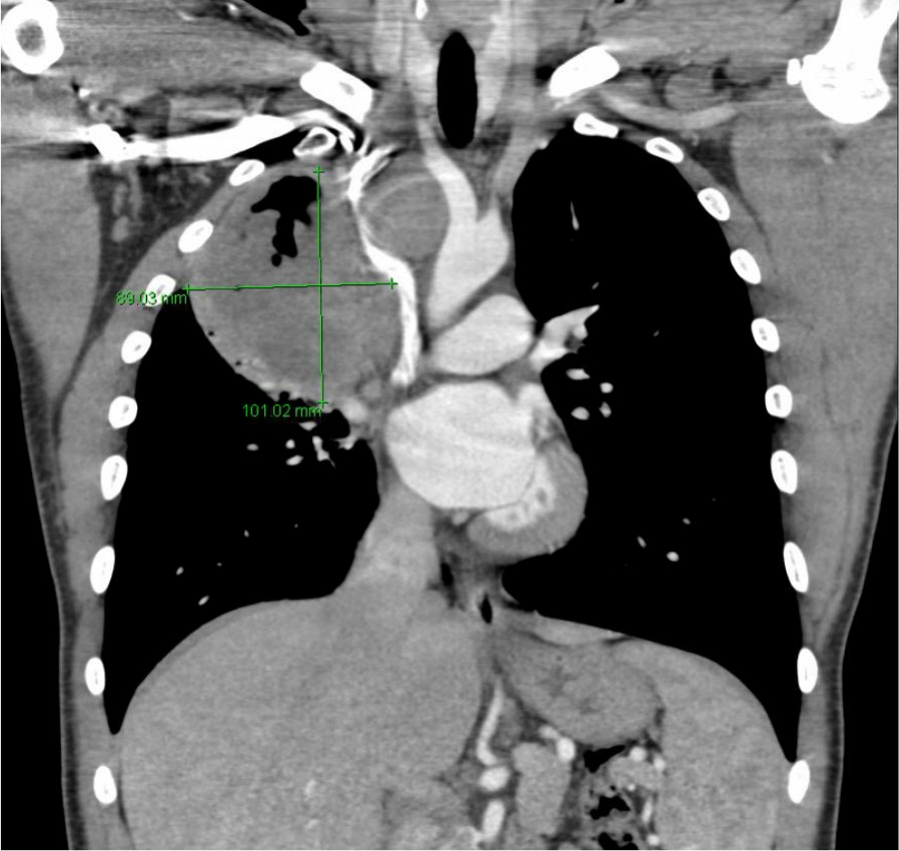
Initial CT chest with contrast (coronal image). Right upper lobe cavitary lesion with compression of the superior vena cava and azygous vein by the and lymph node conglomerate

**Fig. 4:**
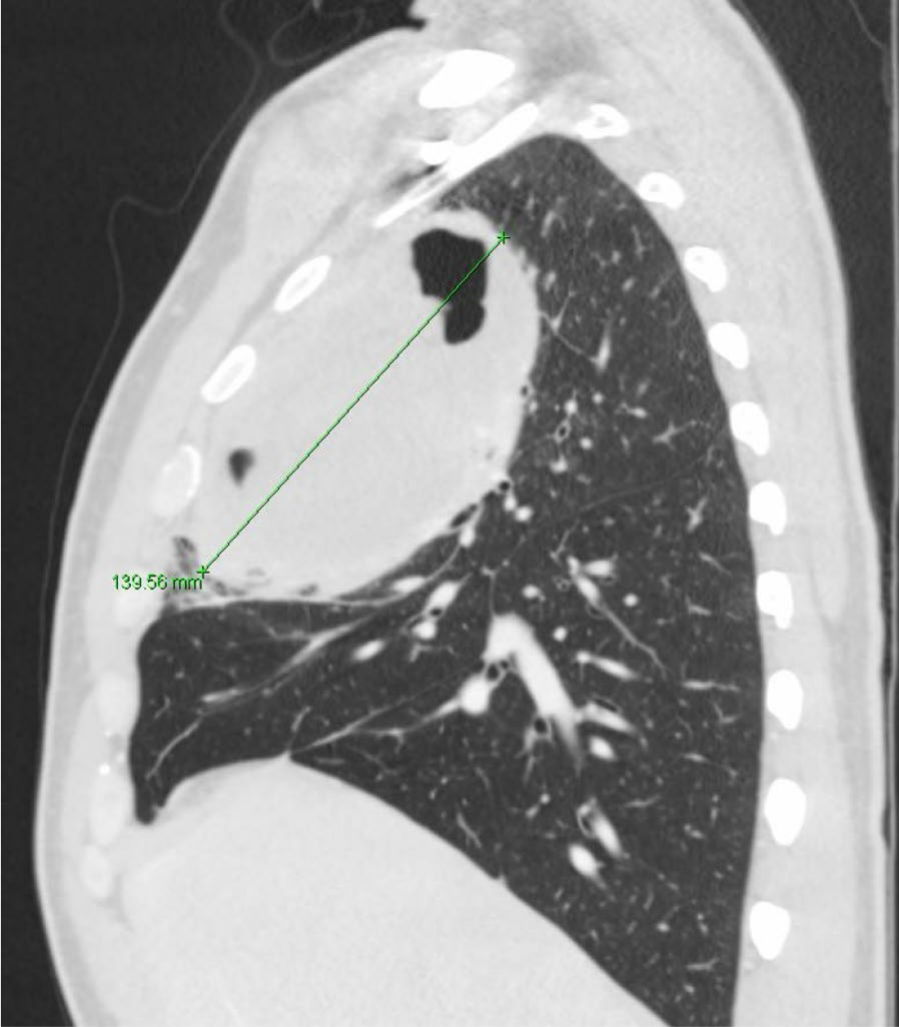
Initial CT chest with contrast (sagittal image). Right upper lobe cavitary lesion

**Fig. 5:**
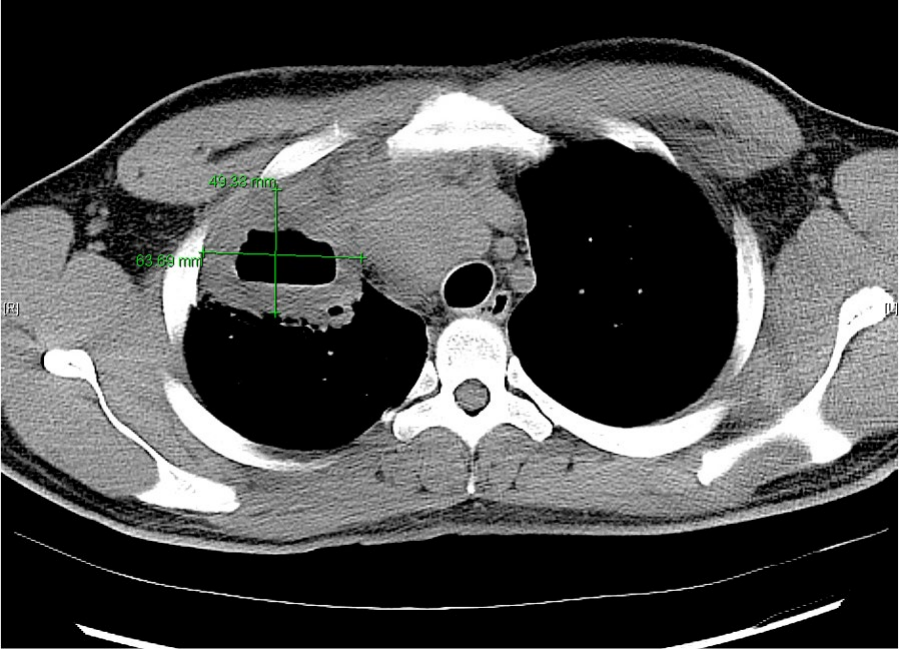
Initial CT chest (axial image). Right upper lobe cavitary lesion

He eventually established with a primary care provider (PCP) and underwent an initial chest X-ray (CXR) that showed a large right upper lobe cavitary lesion (Figs. 1, 2). Further evaluation with computed tomography (CT) of the chest (Figs. 3, 4, 5) demonstrated a right upper lobe 9 × 10 × 14 cm necrotic cavitary lesion with mediastinal adenopathy and compression of the superior vena cava and azygous vein by the right paratracheal extent of the lesion and lymph node conglomerate.

During his initial pulmonary clinic consultation, his physical examination revealed normal vital signs and oxygen saturation and good dentition. He had no oral lesions, facial plethora, jugular venous distention, or cervical or supraclavicular lymphadenopathy. On chest auscultation, his breath sounds were clear bilaterally. He had no palpable lymphadenopathy elsewhere. The remainder of his examination was unremarkable.

**Fig. 6:**
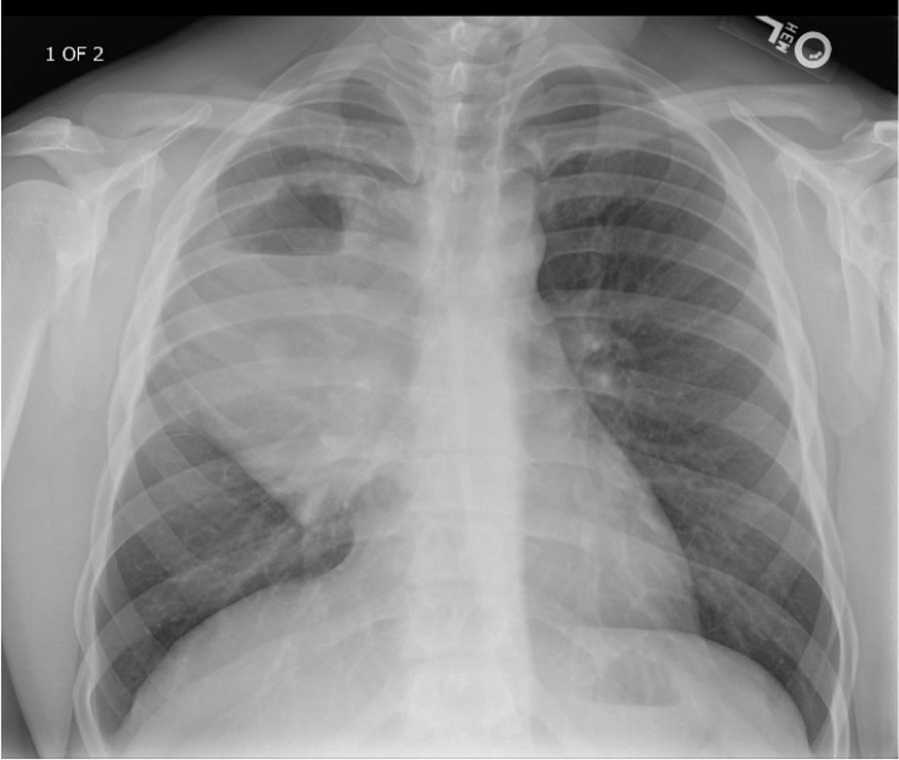
Follow-up chest X-ray (PA view). Enlarging right upper lobe cavitary lesion

**Fig. 7:**
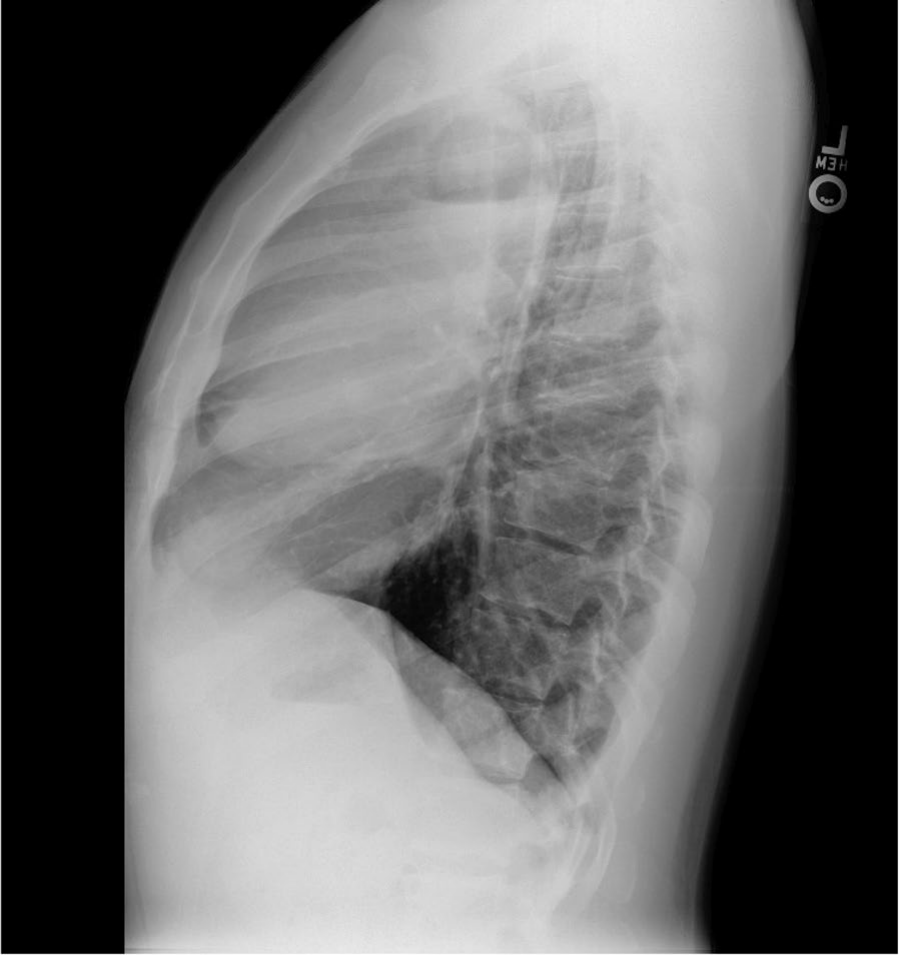
Follow-up chest X-ray (lateral view). Enlarging right upper lobe cavitary lesion

**Fig. 8:**
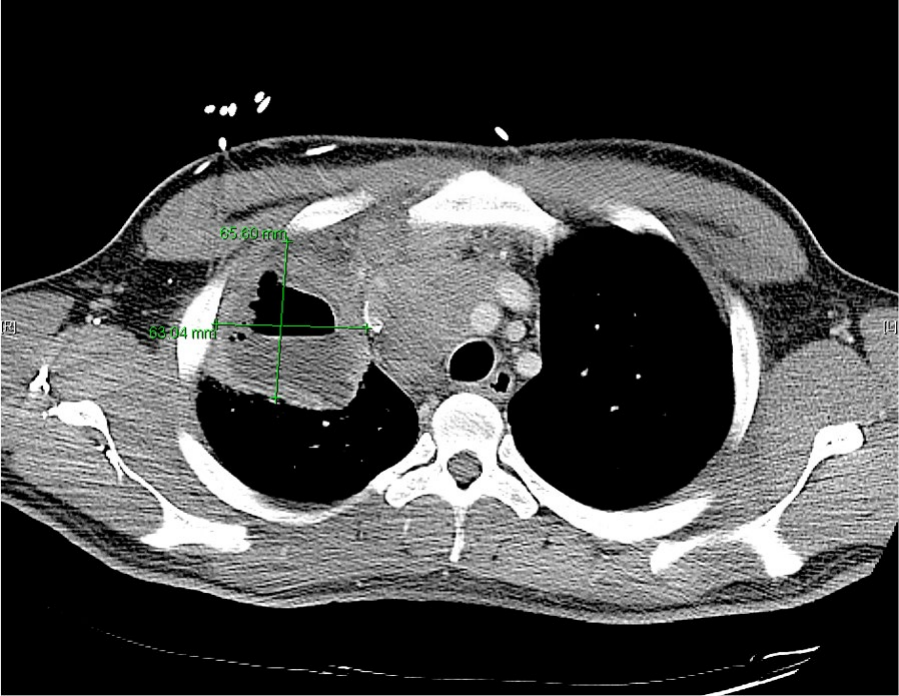
Follow-up CT chest/abdomen/pelvis (axial image). Enlarging right upper lobe cavitary lesion

**Fig. 9:**
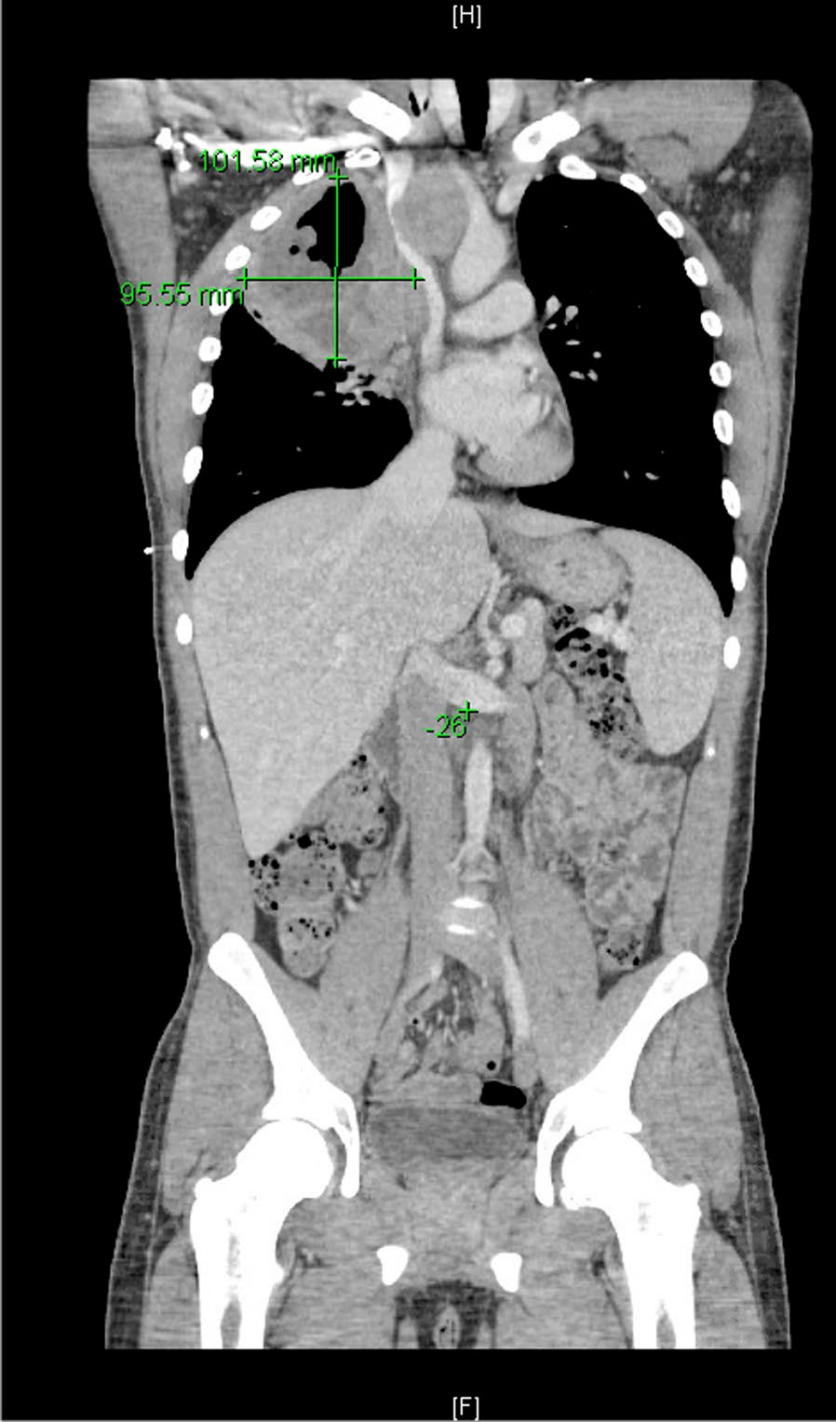
Follow-up CT chest/abdomen/pelvis (coronal image). Enlarging right upper lobe cavitary lesion

**Fig. 10:**
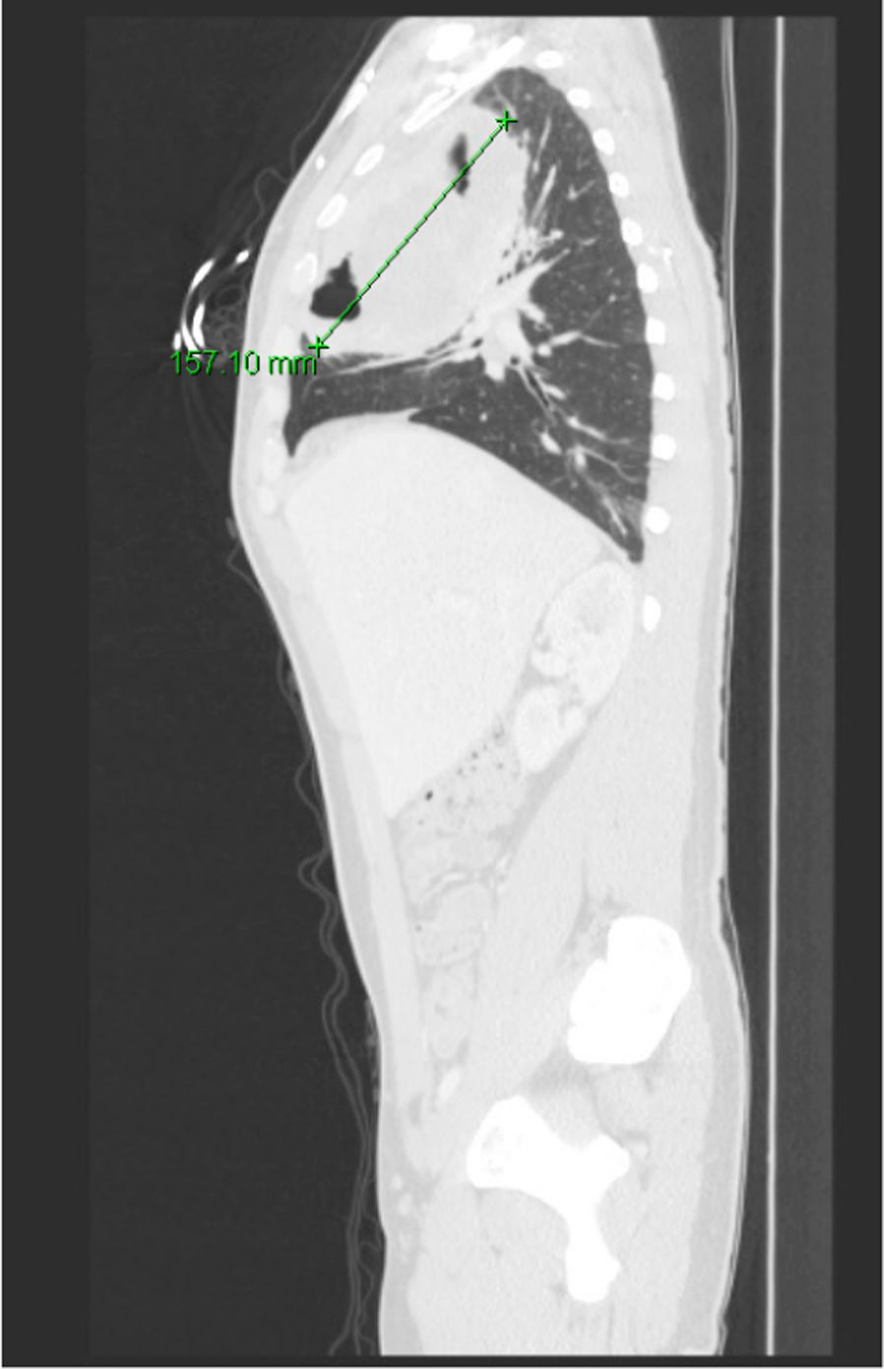
Follow-up CT chest/abdomen/pelvis (sagittal image). Enlarging right upper lobe cavitary lesion

Antibiotic therapy was continued for an additional 2 weeks, first with amoxicillin–clavulanic acid, which was stopped on account of hives, and then with clindamycin. He again experienced mild symptomatic improvement, however, his productive cough returned following completion of therapy. A repeat CXR at 2-week follow-up showed the right upper lobe cavitary lesion had increased in size (Figs. 6, 7). He was then hospitalized to initiate empiric therapy and expedite work-up, including a CT chest/abdomen/pelvis study that demonstrated a larger cavitary pulmonary lesion along with several new smaller cavitary lesions in the right upper lobe (Figs. 8, 9, 10), but no intra-abdominal masses, abdominal lymphadenopathy, or splenomegaly.

Given concern for infection and pulmonary abscess, intravenous antibiotics with clindamycin, vancomycin, and ciprofloxacin were started while awaiting serologic and microbiologic study results. Abnormal laboratory results included an elevated erythrocyte sedimentation rate (ESR) 86, C-reactive protein (CRP) level 13.7, ferritin 835, and d-dimer level 1. Fungal antigen testing revealed negative serum 1-3-β-d glucan, *Aspergillus* galactomannan, cryptococcal antigens, and urinary *Blastomyces* and *Histoplasma* antigens. Bacterial Gram stain and culture of sputum specimen were negative. Acid-fast bacilli (AFB) smears of sequential expectorated sputum specimens were negative. Interferon gamma release assay for *Mycobacterium tuberculosis* was negative with an appropriate mitogen response. His immunoglobulin levels were normal and human immunodeficiency virus (HIV) test was nonreactive. Autoimmune serologies were unrevealing with a negative or normal anti-nuclear antibody test (ANA) panel, anti-neutrophil cytoplasmic antibodies (ANCA), angiotensin-converting enzyme (ACE) level, and rheumatoid factor.

In preprocedural testing prior to tissue biopsy via bronchoscopy and transbronchial biopsy, he tested positive for COVID-19 with the Integrity Laboratories qualitative real-time RT-PCR assay, which detected nucleic acids from SARS-CoV-2 in his nasopharyngeal swab specimen. His acute infection with SARS-CoV-2 prevented bronchoscopy. A CT-guided percutaneous needle biopsy of the lesion was performed by interventional radiology after the patient was intubated, sedated, and placed on mechanical ventilation by anesthesiology in a separate room under negative pressure with all involved healthcare workers observing strict enhanced airborne precautions. The procedure was uncomplicated, and the patient was successfully extubated in a negative pressure room.

**Fig. 11:**
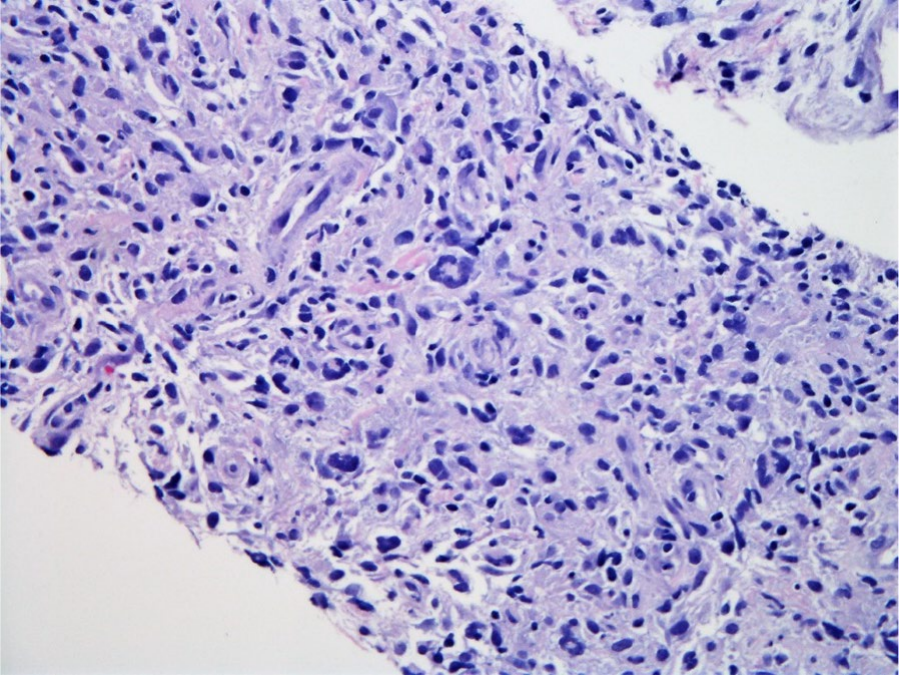
Core needle biopsy specimen showing cellular tissue with large atypical cells in a background of histiocytes, lymphocytes, and occasional plasma cells [hematoxylin and eosin (H&E) stain, 60×]

**Fig. 12:**
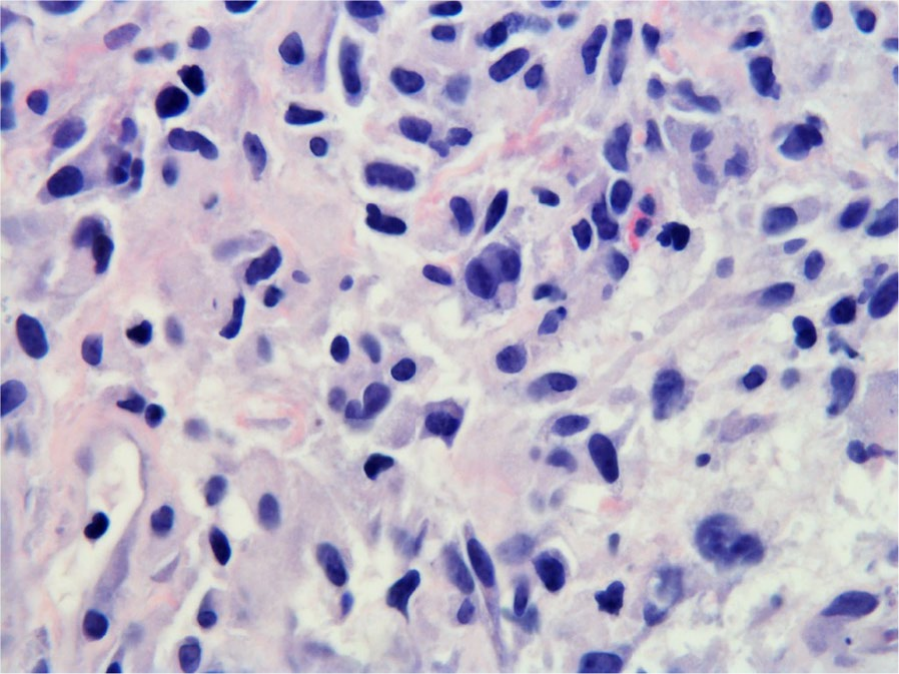
Core needle biopsy specimen showing a binucleated Reed–Sternberg cell residing in an infiltrate of small lymphocytes, eosinophils, and plasma cells (H&E stain, 100×)

**Fig. 13:**
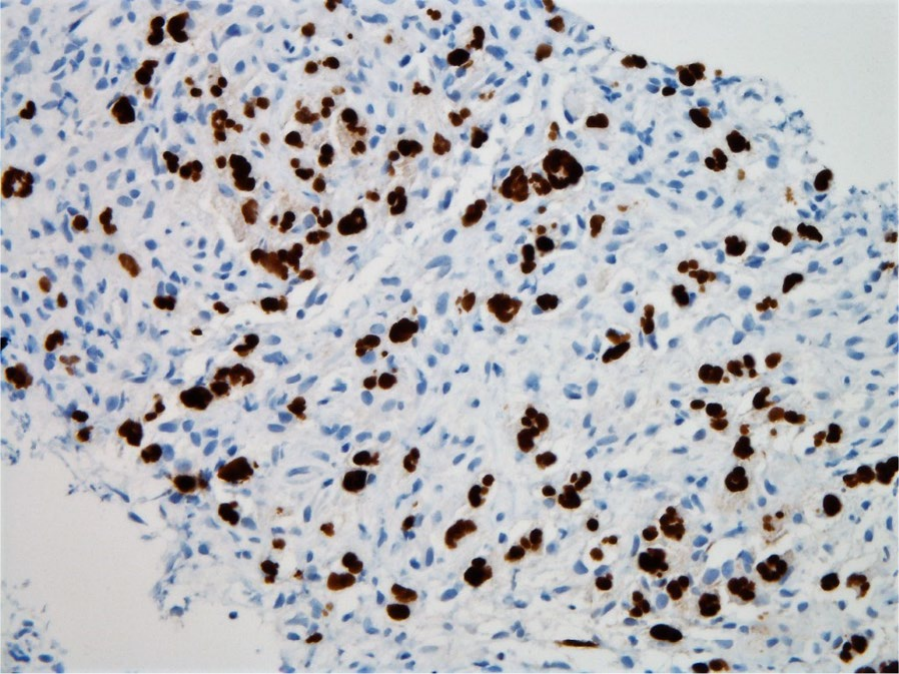
Core needle biopsy specimen with immunohistochemical stains showing positive reactivity for PAX-5 within the large atypical cells (60×)

**Fig. 14:**
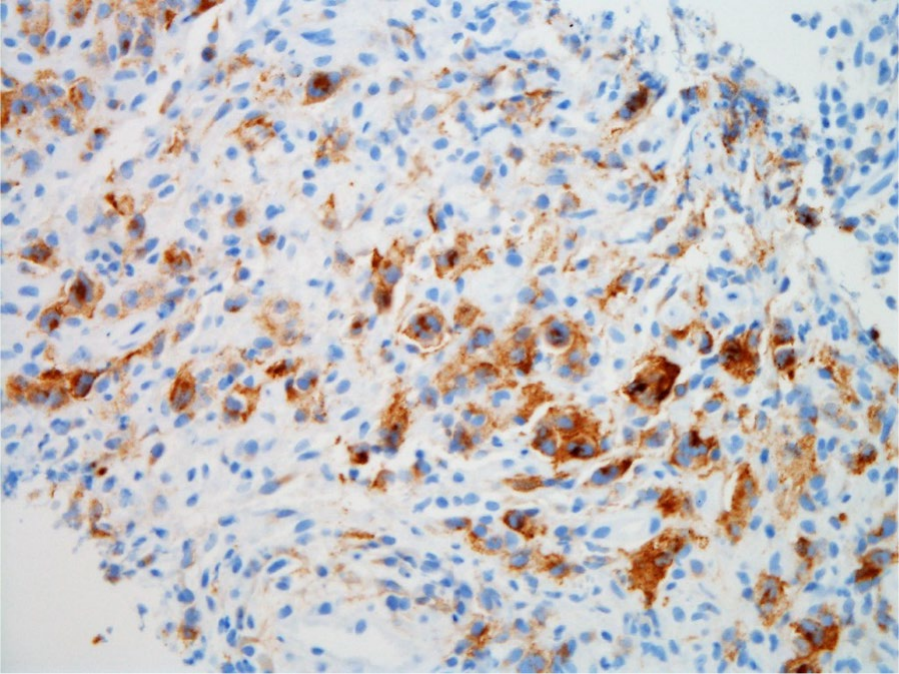
Core needle biopsy specimen with immunohistochemical stains showing positive reactivity for CD30 within the large atypical cells (60×)

**Fig. 15:**
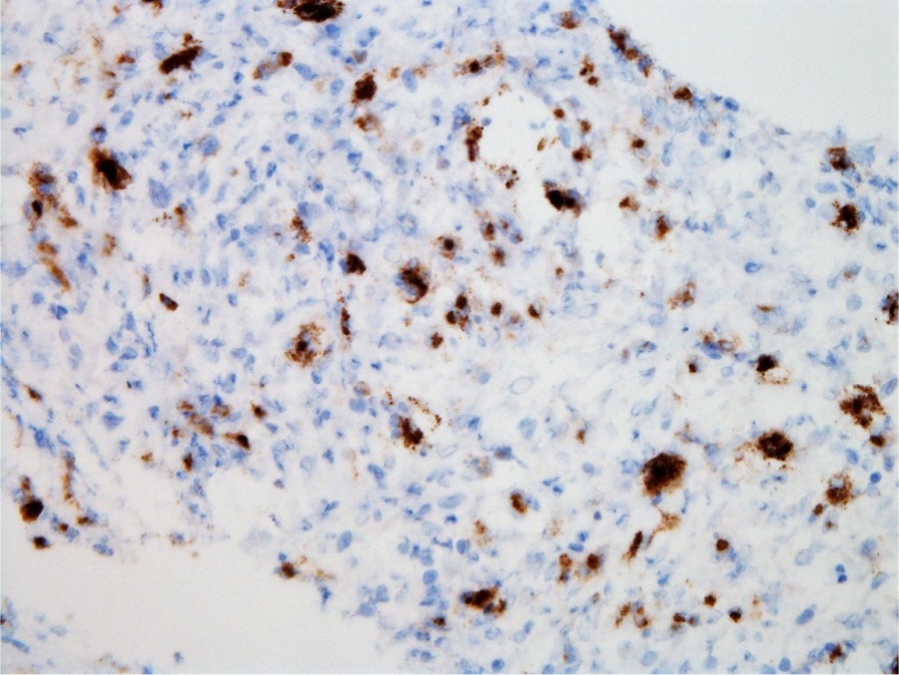
Core needle biopsy specimen with immunohistochemical stains showing positive reactivity for CD15 within the large atypical cells (60×)

Histopathologic evaluation of the core needle biopsy specimen demonstrated classic Hodgkin lymphoma with a mixed cellularity type showing typical Reed–Sternberg cells and immunohistochemical staining positive for CD15, CD30, and PAX-5 (Figs. 11, 12, 13, 14, 15). The AFB and Grocott methanamine silver (GMS) stains were negative for mycobacteria and fungal organisms, respectively.

The patient received deep venous thrombosis (DVT) chemical prophylaxis with subcutaneous low molecular weight heparin injections that were temporarily held before his biopsy procedure. During his hospitalization, he never developed clinical signs or symptoms to indicate a new DVT, venous thromboembolism, or bleeding.

As complications from COVID-19 in the during treatment of his malignancy were feared, medical oncology postponed treatment until the patient tested negative for COVID-19, and he was discharged from the hospital with close follow-up. He continued to test positive for COVID-19 with two additional tests performed 1 and 2 weeks following discharge. As his disease was aggressive, after an empiric quarantine of over 20 days with no worsening of his condition, he initiated treatment with the first of six cycles of combined chemotherapy and immunotherapy under a study protocol including doxorubicin, dacarbazine, nivolumab, and brentuximab vedotin. He eventually tested negative for COVID-19 over 1 month following his diagnosis; and his treatment course remained uncomplicated. An interim positron emission tomography (PET) scan showed a favorable response with decrease in disease burden.

## Discussion

Hodgkin lymphoma (HL), first described in 1832 by Thomas Hodgkin, is an aggressive malignancy arising from B cells within germinal centers and histologically characterized by the presence of Hodgkin and Reed–Sternberg cells [3, 4]. The majority of patients with HL present with nodal involvement; however, there have been reported cases of extranodal disease including within the lungs, liver, gastrointestinal tract, bones, and thyroid [5, 6]. Pulmonary involvement with this malignancy is relatively common, occurring in 15–40% of cases and involving direct parenchymal invasion of hilar disease [7]. Primary pulmonary Hodgkin lymphoma (PPHL) is extremely rare with approximately 100 cases since 1927. The nodular sclerosis type is most frequently observed, occurring in 60–70% of cases, and it predominantly affects young women [8–10].

PPHL has various radiographic findings such as solitary or multiple nodules, consolidations, and ground glass opacities, and often involves the upper lobes [11, 12]. Kern *et al*. described four cases that shared the following features: (1) typical histologic features of HL, (2) disease restricted to the lung or with minimal hilar lymph node involvement, and (3) adequate clinical and/or pathologic exclusion of disease at other sites [13]. A cavitary pulmonary lesion is an uncommon presentation of PPHL [14–17], with our case one of few reported in the literature.

Cavitary pulmonary lesions present with a broad differential diagnosis including life-threatening infections from bacterial, tubercular, and fungal pathogens, malignancies, granulomatous diseases, inflammatory disorders, and inhalational conditions. Given the wide variety of causes for these lesions, obtaining a tissue biopsy via an invasive procedure is paramount, which may include bronchoscopy and transbronchial biopsy, image-guided percutaneous needle biopsy, or video-assisted thoracoscopy surgery (VATS). All these procedures involve risk of aerosolization of microscopic particles from the respiratory tract of the patient.

In the beginning of the COVID-19 pandemic in the USA, non-emergent surgeries and procedures were halted in our institution as the risk for infection with the SARS-CoV-2 virus transmitted through respiratory droplets from aerosol-generating procedures, and disease within exposed health care workers, were believed to be high. Eventually expert opinion guidelines were published addressing bronchoscopy in patients with suspected malignancy in need of urgent tissue diagnosis. While timely performance was advised, provider discretion and consideration of indication, severity of COVID-19 infection, and timing of symptom resolution were emphasized [18]. Delaying bronchoscopy until 2 weeks had elapsed from a negative COVID-19 test was also recommended [19].

Radiologic societal guidelines stated that any potentially aerosol-generating procedure should be done in a negative pressure room with all involved personnel donning appropriate personal protective equipment (PPE) [20]. Additional preprocedural factors to consider included local infection and death rates, PPE obtainability, and negative pressure room and ventilator availability.

Given the seriousness and rapid progression of disease in our patient, timely biopsy for diagnosis was imperative. The risk of viral aerosolization posed by bronchoscopy was deemed excessive. A CT-guided percutaneous needle biopsy was pursued, but could not be safely performed as our interventional suite was not under negative pressure and a high concern existed for viral aerosolization from periprocedural patient coughing. After discussion with radiology and anesthesiology, the patient was electively intubated and sedated in a negative pressure room, and transferred to and from the interventional suite on mechanical ventilation utilizing a closed circuit with appropriate high-efficiency filters to prevent viral aerosolization during the biopsy. This multidisciplinary strategy prevented a delay in diagnosis at our facility, which had the necessary resources to safely perform such a procedure on a patient actively infected with SARS-COV-2.

All patients hospitalized with COVID-19 at our institution early in the pandemic were considered high risk for thrombosis on the basis of reported hypercoagulability [21] and placed on prophylactic dosing of low molecular weight heparin or subcutaneous heparin. Given his compounded risk for thrombosis from suspected malignancy prior to his tissue biopsy, our patient continued prophylactic subcutaneous low molecular weight heparin for the entirety of his hospitalization and did not develop thrombotic or hemorrhagic complications.

Timing of treatment in oncology patients infected with SARS-COV-2 was problematic early in the COVID-19 pandemic. Patients with cancer who had recently been treated with chemotherapy, immunotherapy, radiotherapy, and surgery were reported as having increased risk for severe events complicating COVID-19 infection, including need for intensive care and mechanical ventilation, and death [22–24]. Guidance for clinicians was limited on when to initiate or resume therapy in previously infected patients. Proposed approaches included delaying chemotherapy until the patient was considered clear of infection by World Health Organization and Center for Disease criteria [25], and assessing the chronicity and aggressiveness of the malignancy to determine the timing of therapy [26]. A lower-risk and slower-growing malignancy, such as a chronic hematologic malignancy, could have treatment delayed for up to 3 months, while higher-grade and more aggressive cancers should not.

Following discussion with medical oncology, our patient was observed for nearly 3 weeks with no change in his clinical condition to suggest development of complications from COVID-19. As his PPHL was aggressive, he started treatment with combined chemotherapy and immunotherapy, and suffered no ill effects. Importantly, apart from fever that resolved as an inpatient, and an intermittent cough, he had no clinical signs of serious infection from SARS-CoV-2 (that is, hypoxia) or radiographic evidence of viral pneumonia. The factors involved in deciding when to begin his HL regimen included his limited disease from COVID-19, adequate time allowed to clear his infection, and his high risk of progression if therapy was further delayed.

## Conclusion

Our experience during the current global COVID-19 pandemic details the evaluation and management of a young man actively infected with SARS-CoV-2 presenting with a cavitary pulmonary lesion which on percutaneous needle biopsy revealed primary pulmonary Hodgkin lymphoma. Our collaborative approach sought to timely diagnose and treat an aggressive malignancy of the respiratory tract while using best practices to mitigate the risk of complication in the patient and viral infection in others. In such cases, clinicians should reference all available guidelines and tailor a strategy most appropriate for the patient that utilizes institutional resources while best protecting healthcare workers.

## Data Availability

Not applicable.
